# Supplementary material for: Exploring Omega-3′s Impact on the Expression of Bone-Related Genes in Meagre (Argyrosomus regius)
Source: Biomolecules. 2023 Dec 31;14(1):56. doi: 10.3390/biom14010056 (PMC10813611; doi:10.3390/biom14010056)
Supplement: Supplementary file 1 [file biomolecules-14-00056-s001.zip › biomolecules-2721820-supplementary.pdf]

Supplementary Table S1. Ingredients of the experimental diets

Ingredients, %	Diet M	Diet H
**Fishmeal**	15.0	15.0
Fish protein hydrolysate	10.0	10.0
Squid meal	35.0	35.0
Krill meal	6.0	6.0
Fish gelatin	5.0	5.0
Wheat gluten	5.0	5.0
Wheat meal	2.5	2.5
Vit & Min Premix	3.5	3.5
MSP (monosodium phosphate)	4.0	4.0
Carophyll Pink 10% - Astaxanthin	0.1	0.1
L-Taurine	1.0	1.0
Soy lecithin	5.0	5.0
Vitamin E	0.0	0.1
Fish oil	5.5	4.3
Fish oil DHA70%		1.8
Soybean oil	0.5	
Rapeseed oil	0.5	
Linseed oil	1.2	1.4
Arachidonic acid 40%	0.3	0.3
Total	100.0	100.0

Ingredients of the commercial diet: WINFAST: Squid meal, Krill meal, Fish hydrolysate, fish meal, Wheat gluten, shrimp meal, algal oil, fish oil.
